# Lipidomic characterization of bile and serum reveals an altered lipid landscape in end-stage primary sclerosing cholangitis

**DOI:** 10.1038/s41598-026-45651-6

**Published:** 2026-04-18

**Authors:** Seul Kee Byeon, Saniha Sabu, Pamela S. Tietz-Bogert, Jagadheshwar Balan, Steven P. O’Hara, Nicholas F. LaRusso, Akhilesh Pandey

**Affiliations:** 1 Department of Laboratory Medicine and Pathology, Mayo Clinic, Rochester, MN USA; 2 Manipal Academy of Higher Education, Manipal, Karnataka India; 3 Institute of Bioinformatics, Bangalore, Karnataka India; 4 Department of Medicine, Division of Gastroenterology and Hepatology, Mayo Clinic, Rochester, MN USA; 5 Department of Quantitative Health Science, Mayo Clinic, Rochester, MN USA; 6 Center for Individualized Medicine, Mayo Clinic, Rochester, MN USA

**Keywords:** Mass spectrometry, Untargeted lipidomics, Lysophospholipid, Lactosylceramide, Biliary metabolism, Biochemistry, Diseases, Gastroenterology, Medical research

## Abstract

Primary sclerosing cholangitis (PSC) is a chronic cholestatic liver disease characterized by inflammation and fibrosis of bile ducts, yet its metabolic underpinnings remain poorly defined. Previous lipidomic studies in PSC have focused on either bile or serum, but a systematic comparison across bile, peripheral blood serum and portal vein serum has not been undertaken. We aimed to characterize lipid alterations in these biofluids in patients with advanced PSC to better understand associated metabolic changes. Bile and serum (peripheral and portal vein) samples were collected during transplantation from patients with end-stage PSC (n = 7 and bile; n = 8 for serum) and controls (n = 16 for bile; n = 13 for serum). High-resolution untargeted lipidomics using liquid chromatography-tandem mass spectrometry was performed to investigate biliary and serum lipid alterations in end-stage PSC. More pronounced lipidomic alterations were observed in bile as compared to serum from peripheral or portal vein, with several lipid subclasses showing significant changes in end-stage PSC. The most notable differences included elevated levels of biliary dihexosylceramides, lysophosphatidic acids and triacylglycerol in PSC. Serum from peripheral and portal vein generally exhibited similar trends, with a decrease in lysophospholipids. These findings expand the current understanding of lipid dysregulation in advanced PSC and highlight the distinct metabolic alterations in bile and serum compartments.

**Supplementary Information:**

The online version contains supplementary material available at 10.1038/s41598-026-45651-6.

## Introduction

Primary sclerosing cholangitis (PSC) is a rare yet severe, idiopathic liver disease, resulting from chronic inflammation, fibrotic scarring, and subsequent multifocal stenosis of the intrahepatic and extrahepatic biliary tract^1,2^. The resulting cholestasis leads to hepatic injury, cirrhosis, and end-stage liver disease. Patients have up to 20% lifetime risk of developing cholangiocarcinoma, as well as increased risk of other malignancies including gallbladder cancer, colorectal cancer, and hepatocellular carcinoma^3,4^. Up to 75% of PSC patients have concurrent inflammatory bowel disease (IBD)^5^, which can further exacerbate the risk of developing cancers^4^.

The global incidence of PSC is 0.87 per 100,000 persons per year, with an estimated prevalence of 13.5 per 100,000^6^. Current diagnosis criteria include assessment of clinical features, laboratory investigations, radiographic and histological findings^7^. The overall prognosis for PSC remains poor, with liver transplantation becoming a necessity for patient survival over the course of disease progression^8^.

The molecular mechanisms underlying PSC remain elusive^2^. Alterations in the gut-liver axis, including shifts in gut microbial composition and metabolite flux, are hypothesized to contribute to PSC pathophysiology^9^. This axis is thought to involve the bidirectional communication of metabolites, lipids, cytokines and other molecules between the liver and gut via the biliary tract and portal vein. Such perturbations may influence lipid homeostasis, which is critically linked to inflammation, oxidative stress, metabolic dysregulation, and liver fibrosis^10,11^, although the specific lipid pathways involved in PSC remain incompletely defined.

Previous studies in PSC patients have reported elevated serum lipid levels, which normalize after liver transplantation^12^. Lipidomics and metabolomics approaches have revealed broad alterations in triacylglycerols, lipoproteins, and other metabolites^13,14^. However, these studies focused on broad classes, whereas the lipid groups such as lysophosphatidylinositol (LPI), phosphatidylserine (PS), cardiolipin (CL) and trihexosylceramide (THC) have not been studied, despite their known roles in liver disease and fibrogenesis^15^. Further, bile represents a critical yet underexplored component relevant to hepatobiliary disease and gut-liver interactions^16^ To better characterize lipid alterations associated with PSC , we performed untargeted lipidomics analysis of bile and serum obtained from peripheral blood and portal vein of PSC patients along with non-PSC controls.

## Methods

### Samples

This study was approved by the Mayo Clinic Institutional Review Board (#13-001312). We conducted the study in accordance with the relevant guidelines and regulations outlined in the Declaration of Helsinki. From August 2015 to July 2020, we prospectively enrolled adult participants from the liver transplant inpatient and outpatient services at Mayo Clinic, all of whom provided written informed consent.. Eligible patients were invited to participate when they were scheduled to be the recipient of or donor for a living donor liver transplant. Exclusion criteria included: (i) concomitant liver disease, (ii) prior organ transplant, (iii) renal failure requiring hemodialysis, or (iv) had been treated with an investigational drug or had acute gastrointestinal illness in the previous six months. Charts were reviewed to confirm eligibility and blood from portal and peripheral vein, and bile (~ 2 ml) were collected. The samples were placed on ice, divided into 100 ml aliquots and promptly frozen at −80° C.

### Lipid extraction

Internal standard mixture was prepared by mixing various deuterated mixtures and standards. The UltimateSPLASH ONE lipid standard from Avanti Research (Alabaster, AL), consisting of 69 deuterated species of lipids from 15 subclasses including phosphatidylcholine (PC), phosphatidylethanolamine (PE), phosphatidylglycerol (PG), phosphatidylinositol (PI), PS, lysophosphatidylcholine (LPC), lysophosphatidylethanolamine (LPE), lysophosphatidylglycerol (LPG), LPI, lysophosphatidylserine (LPS), diacylglycerol (DAG), triacylglycerol (TAG), sphingomyelin (SM), ceramide (CER) and cholesteryl ester (ChE), at various concentrations, was diluted 60-fold in methanol containing 1 pmol/µl of d18:1/24:1-dihexosylceramide (DHC) d7, d18:1-d7/15:0-DHC, d18:1-d5/18:0-glucosylceramide (GlcCer), and d18:1-d7 glucosylsphingosine (GlcSph), Cardiolipin Mix I from Avanti Research. Lipids were extracted from 20 µl of serum as described previously^17^. Briefly, an equal amount of a deuterated lipid standard mixture was added to each sample, followed by the addition of an organic solvent mixture (chloroform:methanol, 1:2, v/v). Samples were vortexed and centrifuged to extract neutral lipids, and the resulting organic phase was collected. An acidic extraction was then performed on the remaining pellet using a mixture of chloroform:methanol:37% HCl (40:80:1, v/v/v). After the addition of chloroform and 0.1 M HCl to induce phase separation, the samples were vortexed and centrifuged. The lower (organic) layer was combined with the previously collected organic phase and dried using a vacuum centrifuge. The same procedure was used to extract lipids from bile. For bile samples, 20 µL of the bile supernatant, which was obtained after centrifugation at 10,000 × g for 5 min at 4 °C, was used. The dried lipid extract was reconstituted in 80 µL of methanol:chloroform (1:3, v/v) for subsequent liquid chromatography-tandem mass spectrometry (LC–MS/MS) analysis. All solvents were LC–MS grade and obtained from Fisher Scientific (Waltham, MA), and 37% HCl was purchased from MilliporeSigma (Burlington, MA). All bile samples were prepared together, and all serum samples were prepared together, with each specimen type processed in the same batch.

### Liquid chromatography-tandem mass spectrometry for untargeted lipidomics

Bile and serum lipids were analyzed using untargeted LC–MS/MS. Reconstituted lipid extracts were separated on a Hypersil GOLD Vanquish C_18_ UHPLC column (150 × 2.1 mm, 1.9 μm, 175 Å) using an Orbitrap tribrid IQ-X mass spectrometer (Thermo Fisher Scientific, Waltham, MA) coupled to a Vanquish Flex Binary UHPLC system (Thermo Fisher Scientific, Waltham, MA). A data-driven MS/MS analysis using iterative inclusion and exclusion lists via the AcquireX Intelligent Workflow was employed to generate MS/MS and MS^3^ data, as described previously^18^. A binary gradient was applied at a flow rate of 300 µL/min. Mobile phase A consisted of water:acetonitrile (4:6, v/v) with 10 mM ammonium formate and 0.1% formic acid. Mobile phase B consisted of isopropanol:methanol:acetonitrile (8:1:1,v/v/v) with 10 mM ammonium formate and 0.1% formic acid. Mobile phase B was increased from 20 to 45% over 2 min, to 60% over the next 2 min, to 70% over 6 min, to 85% over 3 min, and to 95% over 4 min. It was then held at 95% for 3 min. Subsequently, mobile phase B was decreased back to 20% over 0.1 min and equilibrated for 5 min before the next injection. The analytical column was maintained at 50 °C. Full-scan MS was performed with a resolution of 60,000 at m/z 200. The precursor mass range was 250–1500 m/z in positive ion mode and 250–1600 m/z in negative ion mode for anionic phospholipids and fatty acids. Dynamic exclusion was enabled with a duration of 5 s. MS/MS spectra were acquired at a resolution of 15,000 at m/z 200. MS/MS scans were followed by a full MS scan.

In positive ion mode, MS/MS fragmentation was performed using stepped collision energies of 25%, 30%, and 35% in higher-energy collisional dissociation (HCD) with a spray voltage of 3.5 kV. Collision-induced dissociation (CID) with a collision energy of 32% was triggered upon detection of a 184 m/z product ion during HCD fragmentation, to enable characterization of phosphatidylcholines^18^. MS^3^ acquisition using CID with a collision energy of 35% was triggered in positive ion mode when a product ion corresponding to the neutral loss of a fatty acyl chain with ammonia was detected, for characterization of TAG species. In negative ion mode, full-scan MS followed by MS/MS acquisition was conducted using a spray voltage of 2.7 kV and stepped collision energies of 30%, 35%, and 40% in HCD. LC–MS grade methanol, acetonitrile, isopropanol, water, and formic acid were obtained from Fisher Scientific (Waltham, MA), and ammonium formate was sourced from MilliporeSigma (Burlington, MA). All samples were analyzed in a randomized order to reduce technical bias.

### Data analysis

The mass spectra files were processed using LipidSearch 5.1.6 (Thermo Fisher Scientific) for lipid annotation and quantification. Lipids were annotated based on precursor ion masses and their corresponding MS/MS spectra matched against the database. The precursor ion mass tolerance was set to 5 ppm, and the fragment ion tolerance was set to 10 ppm. Annotated lipids were quantified by calculating their peak areas using LipidSearch. All lipids were normalized to the peak area of an internal standard with the same head group as the target lipids. For bile samples, lipids were additionally normalized to the protein amount. Total lipid levels were determined by summing the peak areas of individual lipid species sharing the same head group. Statistical significance of levels of total and individual species of lipids between groups was assessed using a Student’s t-test. P values were adjusted using the false discovery rate (FDR) method and Grubb’s test was applied to evaluate the outliers. Principal components analysis (PCA) plots were generated using MetaboAnalyst based on the untargeted lipidomics data. Spearman rank correlation was used to assess associations between lipid levels and the model for end-stage liver disease (MELD) score.

## Results

### Diverse species of lipids are identified from bile and serum samples from patients with PSC

A total of 44 patient biological samples were included for this lipidomic profiling study. Study participant clinical characteristics and biochemical laboratory results are summarized in Table 1. Consistent with gender predisposition and an association with IBD, PSC patients were predominantly male (11/13, ~ 85%) and 9/13 (~ 69%) had concurrent IBD, while 3/13 (23%) also had a history of colectomy. Lipids were extracted from bile samples obtained from PSC patients (n = 7) and controls (n = 16), as well as peripheral and portal vein serum of PSC patients (n = 8) and controls (n = 13). Using untargeted LC–MS/MS, lipids were identified and quantified. A total of 526 biliary lipids across phospholipids (PC, PE, (PG, PI, phosphatidic acid (PA), PS, LPC, LPE, LPG, LPI, lysophosphatidic acid (LPA), LPS, monolysocardiolipin (MLCL) and CL), sphingolipids (sphingoid (SPH), CER, monohexosylceramide (MHC), DHC, THC and SM), fatty acyl lipids (acyl carnitines (AcCa) and free fatty acids (FFA)), glycerides (DAG and TAG) and sterol including ChE were identified and quantified from patients with PSC (n = 7) and controls (n = 16), as listed in Supplementary Table 1. In serum, a total of 586 lipids were analyzed from PSC (n = 8) and controls (n = 13), as shown in Supplementary Table 2. CL and LPS species were detected exclusively in bile, not in serum. Changes in total lipids were evaluated and distinct patterns were observed across bile (Supplementary Table 3), serum from peripheral blood and portal vein (Supplementary Table 4).

**Table 1:** Subject characteristics of controls and patients with primary sclerosing cholangitis (PSC).

	Bile		P-value	Serum		P-value
	Control (n = 16)	PSC (n = 7)		Control (n = 13)	PSC (n = 8)	
Age (years)			0.42			0.31
Median (IQR)	44.8 (36.6–53.3)	40.7 (36.0–53.7)		40.5 (35.5–53.0)	44.3 (39.9–62.0)	
Range	28.3–55.9	28.3–69.4		29.9–55.9	32.6–69.4	
Sex			0.36			< 0.01
Female	8 (50%)	2 (28.6%)		7 (54%)	0 (0%)	
Male	8 (50%)	5 (71.4%)		6 (46%)	8 (100%)	
Body-mass index			0.33			0.57
Median (IQR)	26.6 (23.2–27.9)	23.5 (20.9–23.9)		24.6 (23.8–27.6)	23.4 (21.4–32.3)	
Range	19.7–30.0	19.7–30.9		19.7–29.5	19.7–36.3	
Model for end-stage liver disease score			< 0.01			< 0.01
Median (IQR)	N/A	23.0 (22.0–23.5)		N/A	22 (18–23.5)	
Range	N/A	20.0–31.0		N/A	13–26	
Alkaline phosphatase			< 0.01			0.06
Median (IQR)	62.0 (51.0–68.0)	485.0 (402.0–707.0)		64 (46–70)	263.5 (167–593.25)	
Range	33–109	152–1209		33–109	80–1679	
Alanine aminotransferase			0.11			0.11
Median (IQR)	28.0 (26.8–93.8)	216.0 (118.0–227.0)		27 (26–45)	127 (58.25–222)	
Range	13–209	45–742		17–196	33–623	
Aspartate aminotransferase			0.20			0.07
Median (IQR)	28.5 (20.8–63.0)	150.5 (103.5–214.0)		28 (21–35)	139 (66.5–186)	
Range	17–204	60–884		17–194	29–417	
History of colectomy			0.17			0.08
Yes	0 (0%)	2 (28.6%)		0 (0%)	3 (37.5%)	
No	16 (100%)	5 (71.4%)		13 (100%)	5 (62.5%)	
Inflammatory bowel disease			< 0.01			< 0.01
Yes	N/A	6 (85.7%)		N/A	5 (62.5%)	
No	N/A	1 (14.3%)		N/A	3 (37.5%)	
Pruritus/itching			< 0.01			< 0.01
Yes	N/A	6 (85.7%)		N/A	5 (62.5%)	
No	N/A	1 (14.3%)		N/A	3 (37.5%)	
Diabetes		0.36	0.36			0.35
Yes	0 (0%)	1 (14.3%)		0 (0%)	1 (12.5%)	
No	16 (100%)	6 (85.7%)		13 (100%)	7 (87.5%)	

*IQR* interquartile range.

### Biliary and serum lipids are significantly altered in subjects with PSC

**Fig. 1:**
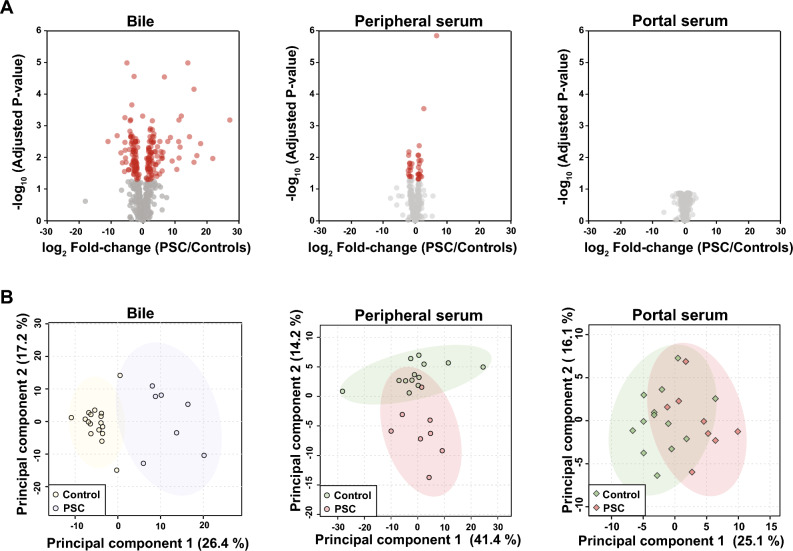
Profiling of lipids from bile and serum in patients with PSC. (**A**) Volcano plots of lipid analyzed from bile, peripheral serum and portal serum. Lipids with significant differences (adjusted P < 0.05) in PSC are marked in red while those without statistical significance are shown in gray. (**B**) A principal component analysis of untargeted lipid profiles in bile and serum, comparing patients with PSC to controls. Each point represents a subject.

Bile lipids exhibited greater alterations in patients compared to those in peripheral and portal serum, with a larger number of lipid species showing significant changes (adjusted P < 0.05) in bile (Fig. 1A). In bile, several subclasses of lipids including DHC, LPA, PA and TAG were significantly increased (P < 0.05), whereas total levels of CER, SPH and LPG were significantly decreased (P < 0.05) as compared to controls. In both peripheral and portal serum, total levels of LPC, LPE and LPI were significantly decreased (P < 0.05) in PSC compared to controls (Supplementary Fig. 1A). Total LPA levels were significantly elevated in bile and peripheral serum from PSC patients and a similar upward trend was also observed in portal vein samples although it did not reach statistical significance (P = 0.7). Changes at the individual lipid species level were further investigated and the most notable differences were observed in bile, where 188 species were significantly altered (adjusted P < 0.05) in PSC, compared to 32 species in peripheral serum (Supplementary Tables 1 and 2). While a total of 105 lipids from portal serum exhibited adjusted P < 0.05, none remained significant after FDR correction. Bile lipid profiles displayed clear group separation in the PCA, while serum lipid profiles showed overlapping distribution between patients and controls (Fig. 1B). In addition, peripheral serum was found to have more pronounced alterations than portal serum.

### Biliary dihexosylceramides and lysophosphatidic acids are highly elevated in PSC

**Fig. 2:**
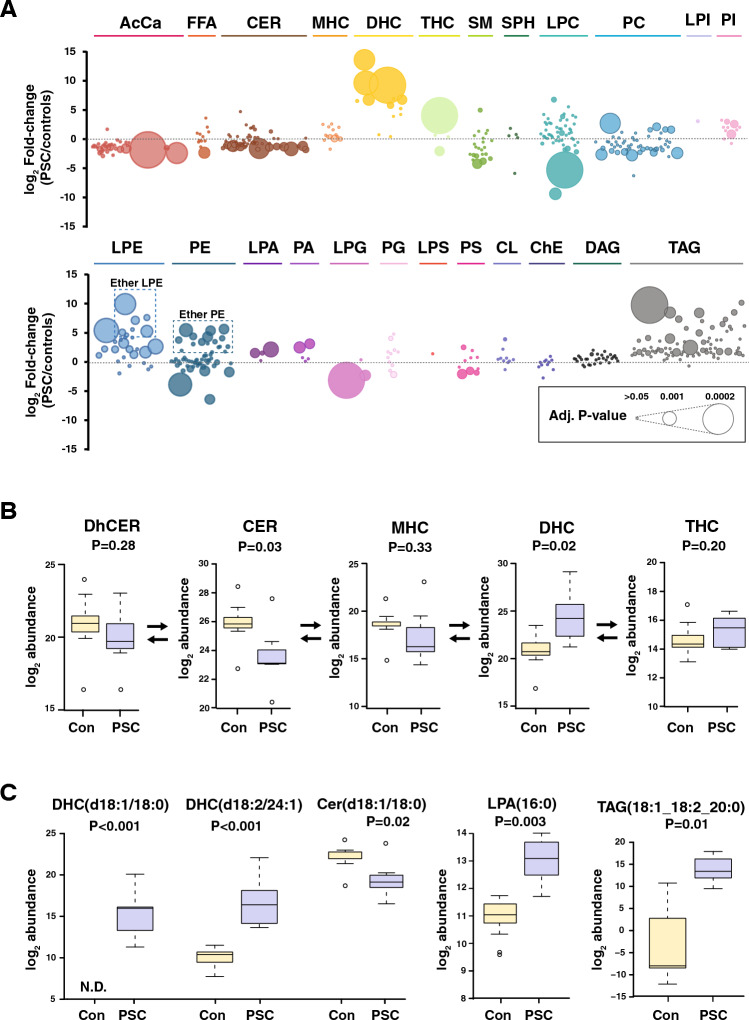
Lipidomic alterations in bile of patients with PSC. (**A**) A scatter plot displaying the average log_2_ fold-change (PSC/controls) in individual lipid species from bile. Each bubble represents lipid species color-coded by lipid subclass, as indicated in the legend above the top panel. Bubble size reflects false discovery rate-corrected P-value, with the scale shown in the box at the bottom. (**B**) Box plots display the abundance levels of dihydroceramide (DhCer), ceramide (CER), and complex glycosphingolipids involved in the sphingolipid pathway, with each box representing values for the corresponding lipid subclasses in patients (yellow) and controls (purple). Arrows between indicate biosynthetic or degradative steps, as indicated by their direction. (**C**) Abundance values in log_2_ scales of select species of dihexosylceramide (DHC), lysophosphatidic acid (LPA) and triacylglycerol (TAG) between patients (red) and controls (green) are illustrated in box plots. *AcCa* acyl carnitine, *FFA* free fatty acid, *CER* ceramide, *MHC* monohexosylceramide, *DHC* dihexosylceramide, *THC* trihexosylceramide, *SM* sphingomyelin, *SPH* sphingoid, *LPC* lysophosphatidylcholine, *PC* phosphatidylcholine, *LPE* lysophosphatidylethanolamine, *PE* phosphatidylethanolamine, *LPA* lysophosphatidic acid, *PA* phosphatidic acid, *LPG* lysophosphatidylglycerol, *PG* phosphatidylglycerol, *LPI* lysophosphatidylinositol, PI phosphatidylinositol, *LPS* lysophosphatidylserine, *PS* phosphatidylserine, *CL* cardiolipin, *ChE* cholesteryl ester, *DAG* diacylglycerol, *TAG* triacylglycerol, *N.D.* not detected.

Broad profiling of lipids revealed distinct clustering between patients and controls in the PCA (Fig. 1B). Many lipid species including DHC, LPE, LPG and LPA species exhibited significant changes exclusively in bile while they remained unchanged in serum (Supplementary Fig. 1A). Ether-linked PE species, where one of the fatty acids is linked to the glycerol backbone via an ether bond instead of an ester bond, were found to be significantly elevated in bile (adjusted P < 0.05), as shown in Fig. 2A. In peripheral and portal serum, although these changes ether PE species showed a decreasing trend in PSC, the changes were not statistically significant (Supplementary Fig. 1B). A total of 99 biliary lipid species (8 DHCs, 1 CERs, 1 THC, 1 SM, 1 DAG, 36 TAGs, 3 LPCs, 5 PCs, 17 LPEs, 12 PEs, 3 LPAs, 2 PAs, 2 PGs, 1 LPI, 3 PIs, 2 PSs and 1 CL) were found to be significantly increased (adjusted P < 0.05) in PSC compared to controls, while 89 species (21 CERs, 3 MHCs, 1 THC, 6 SMs, 16 AcCas, 1 FFA, 6 LPCs, 16 PCs, 11 PEs, 2 LPGs, 3 PGs and 3 PSs) were significantly decreased (adjusted P < 0.05). In bile, sphingolipid metabolism appeared disrupted in PSC, with dihydroceramide (DhCer), CER and MHC showing decreasing trends in PSC, whereas DHC and THC were elevated (Fig. 2B). As shown in Fig. 2C, DHC(18:1/18:0) was exclusively detected in PSC cases and it was not detected in controls. Other DHC species including DHC(d18:2/24:1) were markedly elevated in PSC, showing more than a 50-fold increase with adjusted P < 0.05 (Fig. 2C). In contrast, many species of CER were decreased in PSC by approximately 50% (Fig. 2C). Beyond sphingolipids, several lysophospholipids including LPA, LPE, LPC and LPG, along with TAG species showed significant alterations (adjusted P < 0.05) in PSC (Fig. 2C and Supplementary Fig. 2). Spearman rank correlation was used to assess associations between lipid levels and MELD score, a clinical parameter for severity of liver dysfunction. Among various classes of lipids showing changes in PSC, biliary LPA and ether PE showed positive correlations with MELD score (Supplementary Fig. 3A,B), supporting biological relevance to disease severity.

### Serum lipid profiling reveals changes in peripheral serum in PSC

**Fig. 3:**
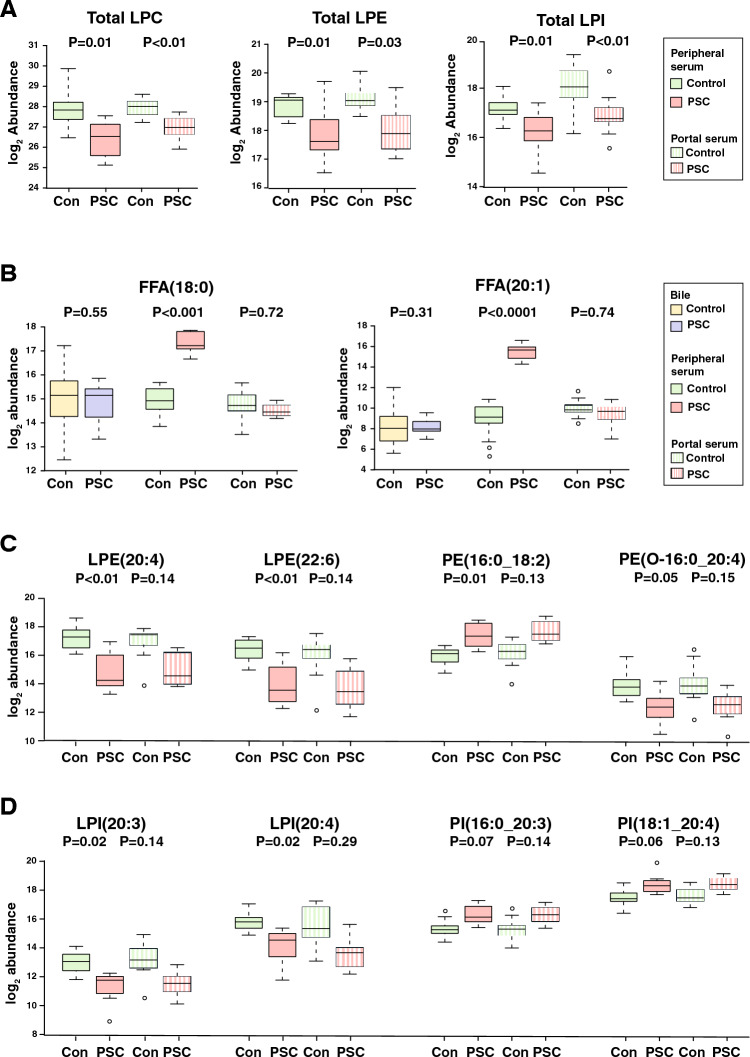
Lipidomic alterations in peripheral and portal serum of patients with PSC. (**A**) Box plots of total lysophospholipid levels (LPC, LPE, LPI) in peripheral and portal serum from controls (green) and PSC patients (red), showing significant changes in patients. (**B**) Box plots of selected free fatty acids (FFAs) in peripheral serum and portal serum (controls: green; patients: red) and bile (controls: yellow; PSC: purple). (**C**) log_2_-transformed abundances of selected LPE, PE, and PE O species in peripheral and portal serum (controls: green; patients: red). (**D**) log_2_-transformed abundances of selected LPI and PI species in peripheral and portal serum (controls: green; patients: red). *LPC* lysophosphatidylcholine, *LPE* lysophosphatidylethanolamine, *LPI* lysophosphatidylinositol, *PE* phosphatidylethanolamine, *PI* phosphatidylinositol.

Lipids were analyzed from both peripheral and portal serum obtained from the same subjects. Similar trends in lipidomic alterations were observed in PSC across both serum types. At the class level, total LPC, LPE, and LPI were significantly reduced in patients (Fig. 3A). At the species level, the most striking changes were observed in two FFAs, FFA(18:0) and FFA(20:1), which were significantly increased by sixfold and 75-fold, respectively, with adjusted P < 0.001. These elevations were unique to peripheral serum, as they remained unchanged in portal serum and bile (Fig. 3B). In peripheral serum, a total of 16 lipids (2 FFAs, 1 AcCa, 9 PEs, 3 PIs and 1 TAG) were significantly increased (adjusted P < 0.05), while 12 lipid species (6 LPEs, 3 LPIs, 1 ether PE, 2 ChE) were significantly decreased in PSC (adjusted P < 0.05). Significant differences were noted in lysophospholipids, with LPE and LPI species showing a consistent decreasing trend (Fig. 3C,D). Although no lipid species from portal serum showed statistical significance after FDR adjustment, the overall directional trends mirrored those observed in peripheral serum. Serum LPE species including LPE(20:4) and LPE(22:6), showed a decreasing trend in both peripheral and portal serum. Similarly, PE species demonstrated an increasing trend in PSC, while ether-linked PEs showed a decreased trend (Fig. 3C). LPI species were consistently decreased, while PI species were elevated in PSC (Fig. 3D). Serum LPE showed an inverse association with MELD score (Supplementary Fig. 3C), suggesting depletion of membrane-derived LPE with increasing disease severity.

## Discussion

We profiled the lipidome from bile, peripheral serum and portal serum in patients with PSC using LC–MS/MS. Distinct lipid alterations were observed between bile and serum, while peripheral and portal serum exhibited similar patterns of change in end-stage PSC. Notably, the elevated levels of biliary DHC, which are known to possess strong pro-inflammatory properties by acting as secondary messengers in the PI3K/Akt signaling pathway, are consistent with findings from a previous study^14^. CER plays an important role in inflammation by promoting apoptosis while inhibiting proliferation^19^. The observed decrease in biliary CER, SM and MHC, alongside increased levels of DHC and THC, suggests alterations in glycosphingolipids synthesis and degradation, which could contribute to inflammation, apoptosis and change in cellular proliferation^20^. These distinct sphingolipids changes were exclusively observed in bile but not in serum. This implies that such changes are likely a result of localized liver or biliary alterations, rather than reflecting systematic changes in circulating serum.

With the untargeted lipidomics approach in this study, we analyzed additional classes of lipids including LPA, PA, LPS, PS, CL and THC that were absent in previous work. In addition to confirming elevated levels of DHCs^14^, we discovered a marked elevation of biliary LPAs in PSC for the first time. Accumulation of LPA has been positively correlated with hepatic inflammation, cholestasis and fibrosis, consistent with the PSC cases used in the study, which are end-stage patients with fibrotic liver^21–26^. As comparable biliary lipidomic data from other chronic cholestatic diseases were not available, we cannot determine whether this increase is specific to PSC or reflects a broader consequence of chronic cholestasis. Notably, among different LPA species, LPA(16:0) has been correlated with liver fibrosis and was significantly increased in PSC bile^23^. Although the increase in LPA species was less pronounced in serum than in bile, an increased trend was observed in both peripheral and portal serum. In contrast, serum levels of LPCs were decreased, differing from the pattern observed in bile. Autotaxin (ATX) converts LPCs to LPAs and studies have reported that increased expression of ATX levels of LPAs in blood are associated with liver injury^27^. In this context, the reciprocal changes in LPCs and LPAs in PSC serum are consistent with altered ATX-related lysophospholipid metabolism within the circulating compartment. LPA and ATX have been implicated in cholestatic pruritus with serum autotaxin activity correlating with itch intensity in adult and pediatric cholestasis^24–26^. In our cohort, pruritus/itch was assessed and total serum LPA levels were not positively associated with the presence of pruritus. For biliary LPA, statistical comparisons between patients with and without pruritus were not feasible due to the limited sample size; however, the patient without pruritus exhibited relatively high biliary LPA levels compared with the rest of the cohort, suggesting that overall increased levels of LPA may reflect disease-related alterations in PSC rather than pruritus.

A significant reduction of LPI species was observed in both peripheral and portal serum from PSC cases. Although not statistically significant, a decreasing trend of biliary LPIs was also noted. LPIs are bioactive lipids that activate G-protein-coupled receptors (GPRs) including GPR55, and are known to influence inflammation, immune regulation and cytokine production through various pathways^28^. The reduction of biliary and serum LPIs observed in PSC may have implications for GPR-mediated signaling and downstream cytokine-associated process profiles, which are hallmark features of PSC pathophysiology, although these pathways were not directly assessed in the present study^29^. Phospholipase A_2_ (PLA_2_) cleaves fatty acids from phospholipids to form lysophospholipids and FFA^30^. The observed reduction in LPIs, alongside increased levels of PIs in both bile and serum may reflect PLA_2_-mediated hydrolysis or altered reacylation of LPIs to PIs occurring within each compartment, although direct relationship between serum or bile lipids were not evaluated. In bile, LPEs were increased while PEs were decreased, reflecting enhanced PE hydrolysis. Conversely, LPEs, which have been reported to be decreased in plasma in advanced cirrhosis, were also decreased in both peripheral and portal serum in our results, while PEs were increased in PSC^31^. These are consistent with prior evidence that PLA_2_ activity varies across different phospholipid subclasses and biological samples, potentially reflecting the presence of PLA_2_ isoforms with different substrate preferences and activities depending on their localization^30^.

Biliary TAGs were also highly elevated in PSC bile. Notably, around 50% of the total elevated TAG species identified had at least one C16 fatty acid while around 80% of the total TAG had at least one C18 fatty acid. The accumulation of TAG especially containing these two fatty acyl chains is significant as they are known to be toxic and pro-inflammatory. Since mitochondrial B oxidation could be compromised, an alternative pathway to sequester the excess FFAs is their utilization in TAG and the formation of lipid droplets^32^. Although not statistically significant, serum TAGs showed an increasing trend in PSC. Increased LPA levels, as observed in PSC patients within bile, as well as peripheral serum and portal serum, can also promote TAG biosynthesis^33^.

The decreased ether-linked PE levels including PE(O-16:0_20:4) in peripheral and portal serum are further indicative of pathological changes in the liver. Ether PE form integral parts of cell membrane and confer protection from oxidative stress. They have been shown to protect against metabolic dysfunction-associated steatotic liver disease by activation of peroxisome proliferator-activated receptor alpha, which can in turn promote fatty acid oxidation. Thus, the reduced levels of ether PE and reduced FFA oxidation may increase hepatotoxicity^34^.

As samples were obtained at the time of liver transplantation, an important consideration was whether the observed lipid alterations are PSC-specific or more general features of advanced liver disease. To address this, we examined associations between bile lipid levels including CL, ether PE, PS and PI and clinical disease severity marker such as MELD score within PSC patients (Supplementary Fig. 3A,D–F). Alterations in cardiolipin content and remodeling have been linked to mitochondrial dysfunction, oxidative stress, and progression of chronic liver disease^35^. Remodeling of phospholipids have also been linked to fibrotic liver injury and pathology^36–38^. These lipid classes showed positive associations with MELD score, indicating biological relevance to disease severity, although they are not necessarily PSC-specific. Given the close association between PSC and IBD, we also evaluated whether lipid alterations differed according to IBD status or history of colectomy. Although no statistically significant differences were identified in this cohort, due to limited sample size in this study, larger studies will be required to determine whether these clinical phenotypes modulate lipid alterations in PSC.

Limitations of this study include incomplete matching of bile and serum from the same individuals and relatively small sample size. As a result, although bile and serum lipid alterations are discussed within a shared biological context, this study could not evaluate direct mechanistic gut-liver axis relationships between compartments. In addition, all patient samples were from the patients with end-stage PSC undergoing liver transplantation. Advanced cirrhosis, chronic cholestasis and hepatic failure are themselves associated with profound metabolic remodeling, which may contribute to or amplify the lipid alterations observed here^33,39,40^. Because samples from earlier stages of PSC and disease control cohort including other chronic cholestatic diseases such as primary biliary cholangitis were not available, the specificity of lipid alterations to PSC could not be assessed. Accordingly, our findings should be interpreted as characterizing lipidomic remodeling associated with end-stage PSC rather than definitive disease-specific biomarkers. These limitations should be addressed in future studies with larger and fully matched cohorts.

## Supplementary Information

Supplementary Information 1.

Supplementary Information 2.

Supplementary Information 3.

## Data Availability

Data is provided within the manuscript.
