# Supplementary figures and images for: Lipidomic characterization of bile and serum reveals an altered lipid landscape in end-stage primary sclerosing cholangitis
Source: Sci Rep. 2026 Apr 18;16:18066. doi: 10.1038/s41598-026-45651-6 (PMC13253874; doi:10.1038/s41598-026-45651-6)

# Supplementary Figure 1

**A**

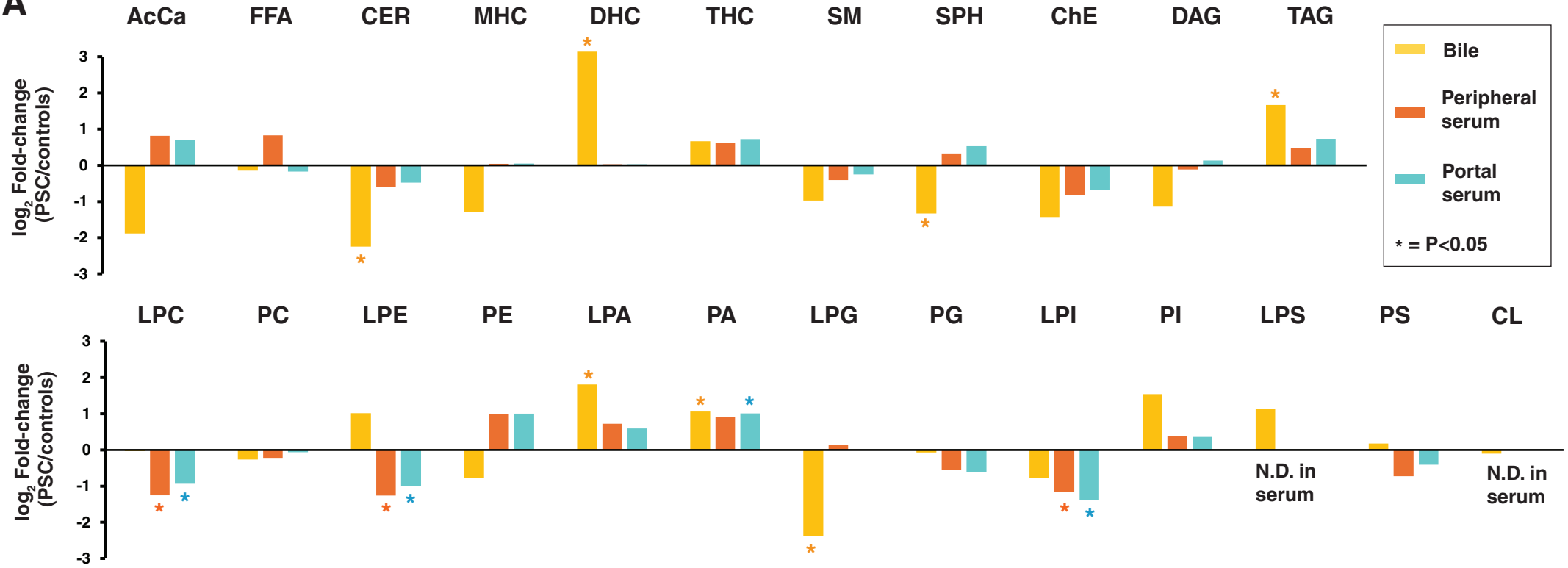

**B**

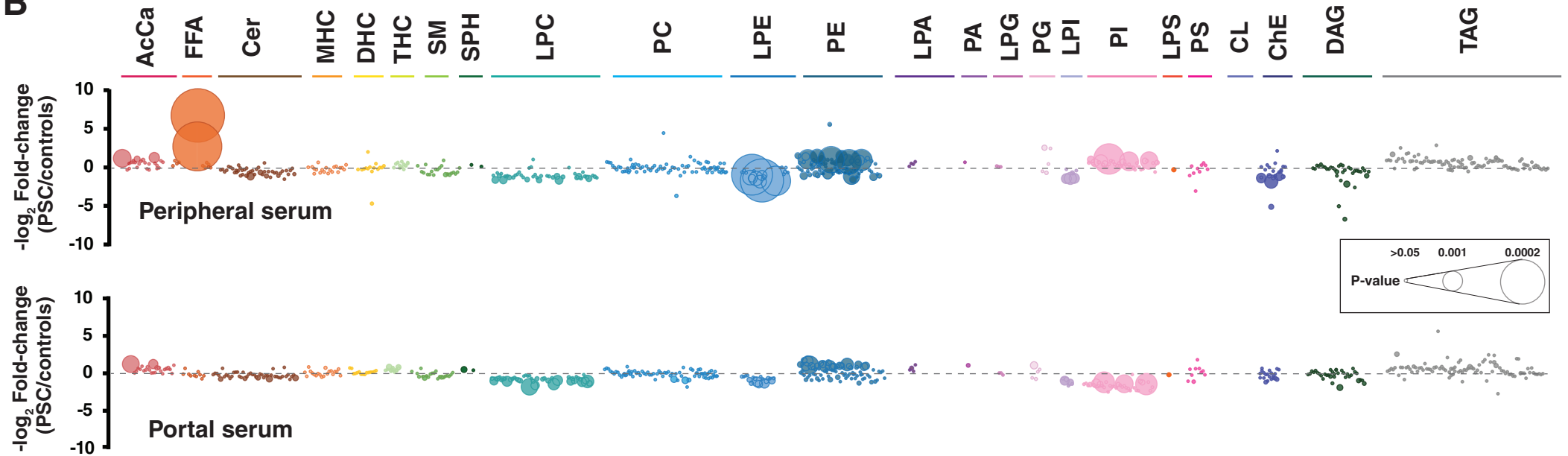

## Supplementary Figure 2

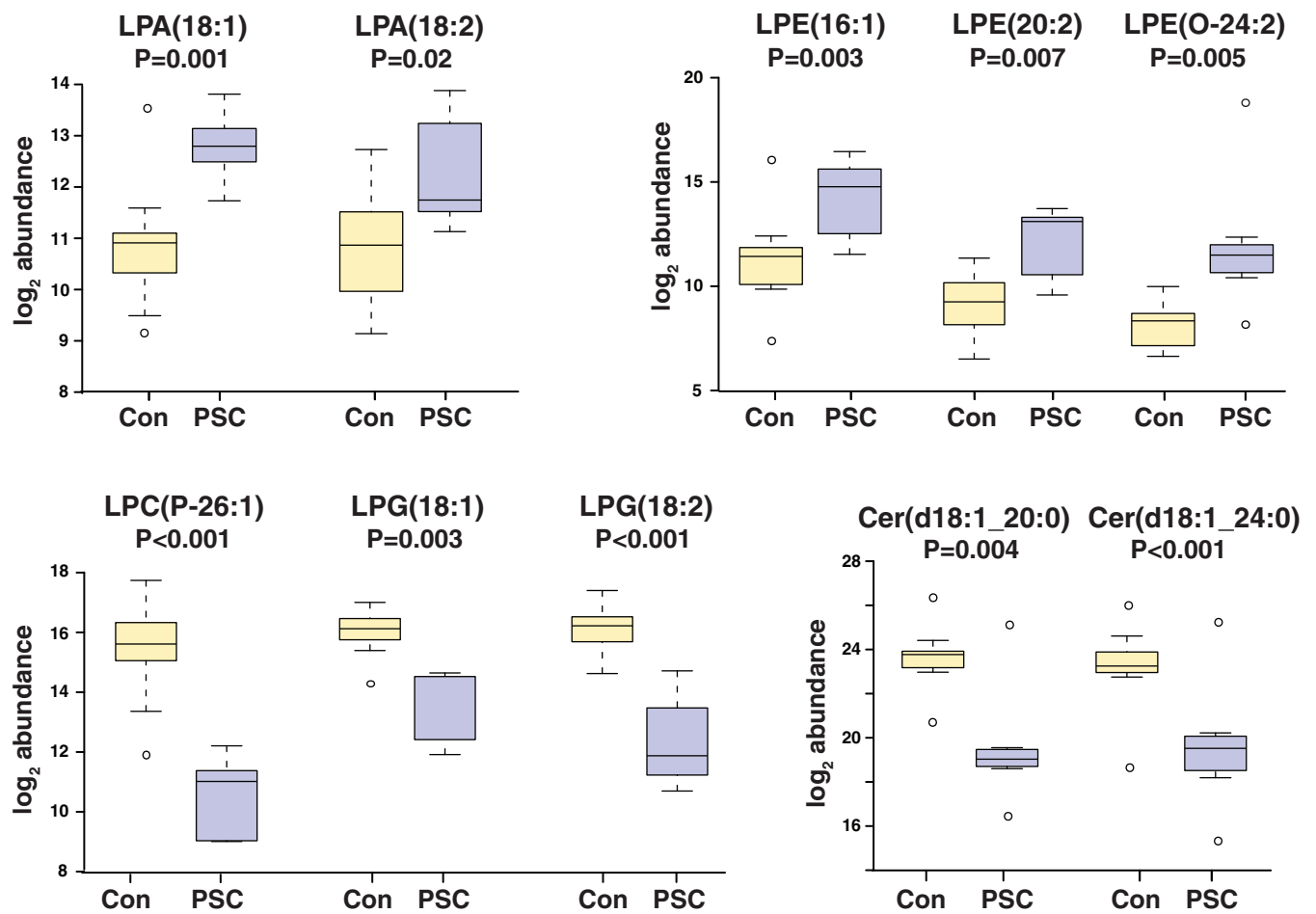

# Supplementary Figure 3

**A**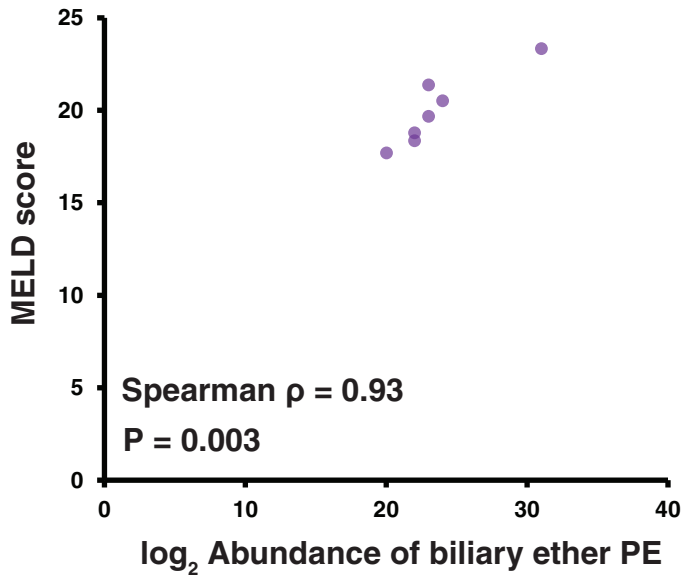**B**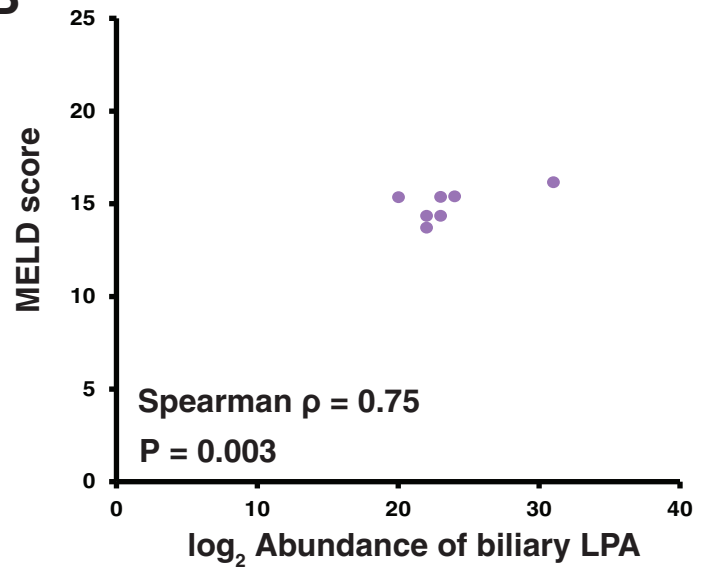**C**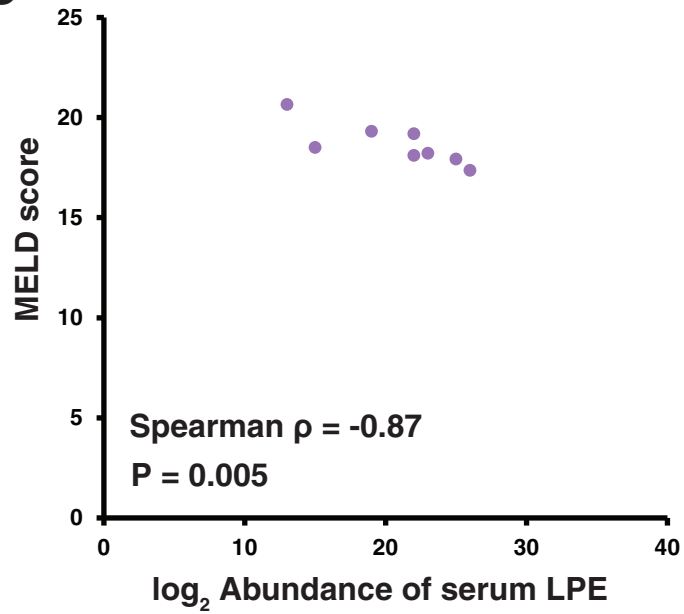**D**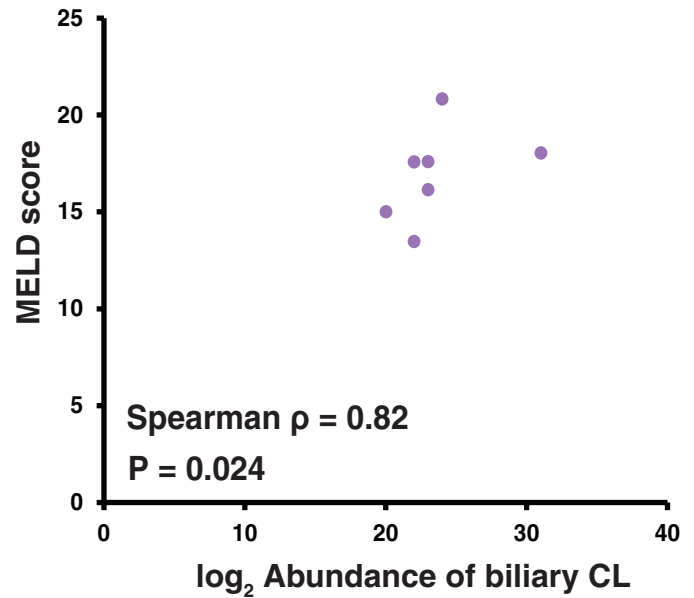**E**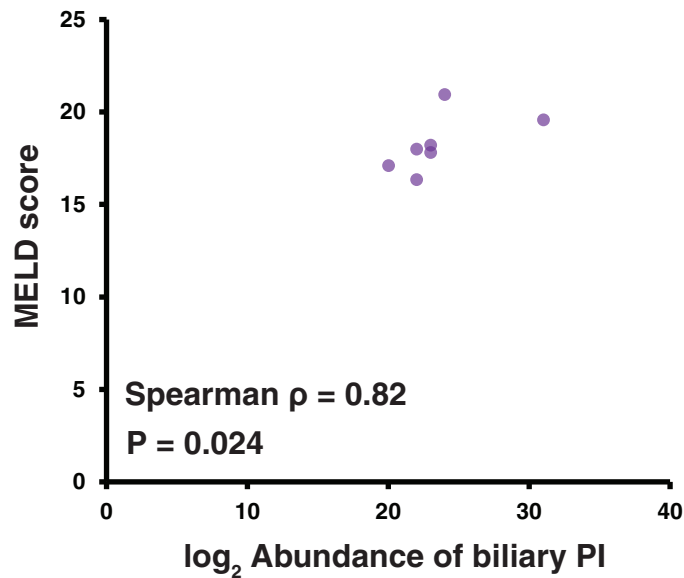**F**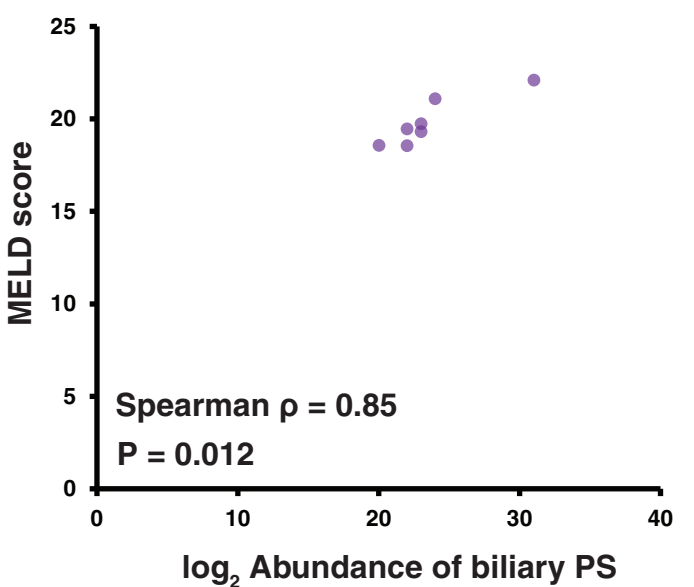

Supplement: Supplementary file 1 — Supplementary Information 1. [file 41598_2026_45651_MOESM1_ESM.pdf]
