# Supplementary material for: Lipidomic characterization of bile and serum reveals an altered lipid landscape in end-stage primary sclerosing cholangitis
Source: Sci Rep. 2026 Apr 18;16:18066. doi: 10.1038/s41598-026-45651-6 (PMC13253874; doi:10.1038/s41598-026-45651-6)
Supplement: Supplementary file 3 — Supplementary Information 3. [file 41598_2026_45651_MOESM3_ESM.docx]

**Supplementary Fig. 1. Quantitative analysis of biliary and serum lipids in patients with PSC.** (A) A bar graph illustrates the average log_2_ fold-change (PSC vs. controls) in total levels of 24 subclasses of lipids. Bile (yellow), peripheral serum (orange) and portal serum (blue) lipid levels are represented by color. Statistically significant differences (P<0.05) are marked with asterisk. (B) A scatter plot displays the average log_2_ fold-change (PSC vs. controls) in individual lipid species from peripheral serum (top panel) and portal serum (bottom panel). Each bubble represents lipid species and is color-coded by lipid subclass, as indicated in the legend above the top panel. Bubble size reflects false discovery rate-corrected P-value, with the scale shown in the box on the right.

*Abbreviations: AcCa=acyl carnitine; FFA=free fatty acid; CER=ceramide; MHC=monohexosylceramide; DHC=dihexosylceramide; THC=trihexosylceramide; SM=sphingomyelin; SPH=sphingoid; LPC=lysophosphatidylcholine; PC=phosphatidylcholine; LPE=lysophosphatidylethanolamine; PE=phosphatidylethanolamine; LPA=lysophosphatidic acid; PA=phosphatidic acid; LPG=lysophosphatidylglycerol; PG=phosphatidylglycerol; LPI=lysophosphatidylinositol; PI=phosphatidylinositol; LPS=lysophosphatidylserine; PS=phosphatidylserine; CL=cardiolipin; ChE=cholesteryl ester; DAG=diacylglycerol; TAG=triacylglycerol; N.D.=not detected.

**Supplementary Fig. 2. Comparison of lipid species from bile between patients with PSC and controls.** Abundance values in log_2_ scales of select species of lysophosphatidic acid (LPA), lysophosphatidylethanolamine (LPE), lysophosphatidylcholine (LPC) and ceramide (CER) are shown in box plots. For all box plots shown in this Fig., upper and lower whisker represent data points that are less than 1.5 times the interquartile range away from third and first quartile, respectively. Open circles represent the outliers beyond this range. The top of the box represents 3^rd^ quartile while the bottom of the box represents 1^st^ quartile. Black line inside the box indicates median value of each group.

**Supplementary Fig. 3 Correlation analysis for association of lipid levels to model for end-stage liver disease (MELD) score.** Scatter plot showing Spearman rank correlation between MELD score and log-transformed (A) biliary ether phosphatidylethanolamines (PE), (B) biliary lysophosphatidic acids (LPA), (C) serum lysophosphatidylethanolamines (LPE), (D) biliary cardiolipins (CL), (E) biliary phosphatidylinositol (PI) and (F) biliary phosphatidylserine (PS).
